# Reflective Functioning of Refugee Mothers with Children Born of Conflict-Related Sexual Violence

**DOI:** 10.3390/ijerph17082873

**Published:** 2020-04-21

**Authors:** Kimberley Anderson, Elisa van Ee

**Affiliations:** 1 Psychotraumacentrum Zuid Nederland, Reinier van Arkel Groep, 5211 LJ ‘s-Hertogenbosch, The Netherlands; e.van.ee@reiniervanarkel.nl; 2 Department of Medical Psychology & Medical Sociology, University of Leipzig, 04103 Leipzig, Germany; 3 Behavioural Science Institute, Radboud University, 6525 HR Nijmegen, The Netherlands

**Keywords:** sexual violence, refugee, reflective functioning, attachment, PDI-RF

## Abstract

The ability of a parent to step back from their own experiences in order to understand those of their child, reflective functioning (RF), can be impacted by myriad factors. We explored RF among refugee mothers in the context of having a child born of sexual violence (CBSV). A sample of 10 mothers now residing in the Netherlands, both with (*n* = 5) and without (*n* = 5) a CBSV, were interviewed, seeking to explore parents’ representations of their children, themselves as parents, and their relationship with their children. After deriving a score of RF, interview narratives were qualitatively analyzed using thematic analysis. An ordinary level of reflective functioning was identified in this sample overall (average score 4.5); which was reduced in the group with CBSV (average score 3.0). Trends within the qualitative analyses indicated that emotion regulation and ambivalence as well as parenting challenges are factors that affect RF capabilities for mothers with CBSV. Wider findings show that the asylum process and mental well-being also impinge on RF capabilities. Experiences of having a CBSV as well as those pertaining to being a refugee appear to interact and impact reflective functioning for some mothers. Further investigation would add weight to this pilot data.

## 1. Introduction

The past two decades have seen increasing recognition of the importance of attachment representations between caregivers and their children. In particular, Fonagy and colleagues have been pivotal in shedding light onto the concept of mentalization—“the process by which we make sense of each other and ourselves, implicitly and explicitly, in terms of subjective states and mental processes” [1] (page 11)—and the capacity of parents to engage in mentalization in order to understand their children [2]. Based on a mother or father’s own attachment history, these same authors describe broadly opposing parenting styles: sensitive parents who perceive their own actions and the actions of others in terms of mental states are more likely to accurately discern and respond to the causes of their child’s distress and proximity-seeking, whereas parents with a limited capacity to mentalize may not accurately recognize their child’s distress, and thus reinforce undesirable behavioral strategies used to invoke a response from the parent. At as young as three, typically developing children are believed to understand that other people have feelings and thoughts as different from their own [3], to which they increasingly learn how to assess and respond. By this distinction, only when a child can reasonably assume that his or her mental state will be accurately responded to will they feel secure in relation to their caregiver. The combination of the parent’s mentalization capacity and sensitivity and the child discovering their own mind through their caregivers’ response to their needs is thought to be at the core of secure attachment relationships [4].

The ability of a parent to step back from their own experience and seek to understand that of their child is termed reflective functioning (RF) [5]. Securely attached adults possess the capacity for reflective functioning in two directions: retrospectively with regard to their childhood and attachment to their own parents, and in terms of their attachment to their own children now they are parents themselves [6]. As a result, Fonagy and colleagues [2] demonstrated that adults who rated themselves as securely attached (even when assessment took place before the birth of a child) also had children who were more likely to be securely attached. Conversely, caregivers who have experienced loss or trauma that remains unresolved can exhibit behaviors that are disturbing to children, and they become at the same time the source and solution of the child’s distress [7].

Loss and trauma are examples of critical experiences that impact the journey of parenting and can be particularly present for parents who are refugees and have histories of migration, where in such situations, women and children are often the most vulnerable [8]. For example, they may experience war both directly and indirectly, travel treacherous migration routes, seek asylum in unknown places, and face assimilation into a new culture [9,10]. While seeking asylum, refugees are continually exposed to daily stressors (e.g., the asylum procedure, housing, financial strains), which may negatively impact the level of parenting sensitivity [11,12]. Intrusive and avoidant trauma-related symptoms are thought to constitute a continuing state of fear [13] and increased stress levels, resulting in a greater production of cortisol that can have lasting epigenetic effects on the unborn child [14,15]. Once the child is born, experiences of trauma can impact the ability of the mother to bond with an infant [4]. This can result in difficulty regulating and controlling emotions and moderating expressions of anger, which can lead to self-harming, suicidal, impulsive, or risk-related behaviors as a reaction to trauma [16]. The effects of trauma on the mother (e.g., avoidance, withdrawal) and the consequences of these effects on the child (e.g., agitation, excitability) may contribute to dysfunctional cyclical interactions, poor child functioning, and insecure parent–child relationships [15,16,17]

Mothers who experience conflict-related sexual violence (CRSV) and give birth to a child (herein referred to as children born of sexual violence, CBSV) often experience myriad psychological disturbances given the physical, relational, and psycho-emotional disturbances following this type of violence, which can impact a child’s early development [18]. Owing to the circumstances surrounding the conception of the child, the trauma itself can impact the connectedness of mother to child, and as a result the capacity for optimal parenting is likely to be challenged. In some cases, mothers are known to experience ambivalence towards their CBSV [19], oscillating emotions and feeling states, and the experience of both positive and negative emotions simultaneously. In more extreme cases, the child may also become the object of violent outbursts if he or she is identified with the aggressor [15]. These types of mental health concerns in mothers are risk factors for decreased functioning in children, particularly regarding anxiety, depression, low self-esteem, attentional disturbances, and adaptive functioning. In addition, stigmatization around CRSV and isolation from their families and communities are frequently realities in their country of origin and prevent women from revealing their experiences [20]. This ostracisation can be a factor contributing to their fleeing and seeking asylum elsewhere [21]. As a result, mothers raising their child outside of their social environment could be said to be one of the most at-risk groups for future parenting challenges.

Therefore, the aim of this study is to explore RF in trauma-affected mothers now residing as refugees in the Netherlands, discerning in particular the capacities of mothers with CBSV. We also hope to utilize the narratives collected during interviews to take a qualitative approach to understanding their individual experiences. It is hypothesized that mothers with CBSV will have lower RF scores than other mothers, but there are likely to be overlapping experiences pertaining to the asylum process. To the best of the authors’ knowledge, this has not been investigated within such a population, and this preliminary work is hoped to provide a basis for future exploration of the needs of this population.

## 2. Materials and Methods

### 2.1. Design and Setting

This cross-sectional study took place within a community mental health facility (Psychotraumacentrum Zuid Nederland, PTC), which is primarily a service for refugees, asylum seekers, and military and police veterans. The PTC is a recognized knowledge center in the south of the Netherlands (NL), combining research with practice. This study was approved by the Dutch Medical Ethical Committee (METC) at University Medical Centre, Utrecht. In the Netherlands, anyone seeking asylum—including those who are undocumented—can access basic mental and physical healthcare.

### 2.2. Participants and Procedure

This study employed a purposive sampling technique to reach refugee women. This involved contacting various institutions across the Netherlands where women, mothers and children, and/or refugees are supported, to gain access to potential participants. Recruitment methods also included advertisements in refugee centers, shelters, safe houses, and clinics. All recruitment took place between May 2016 and December 2017. To reflect the impact of conceiving and raising a CBSV, the study also comprised a “control” group of mothers who had either experienced CRSV but did not conceive a child as a result, and mothers who had experienced other war-related trauma.

Once potentially eligible participants had been identified, they were approached by either their therapist/case worker or the first author with detailed information about participation in this study. After a period of consideration (at least a week), potential participants were contacted again (either face-to-face or via telephone), and individuals who agreed to continue were requested to provide written consent, either with or without the aid of an interpreter, per the choice of the participant. Assessments were carried out in two or more sessions, to account for the emotional burden of such research and the overall level of concentration. All assessments were carried out in either the participant’s home or at the PTC, as per the request of the participant, in the language they preferred. Ten women were included in the final sample (*n* = 5 in each subgroup); a further five women declined participation after having read the study information. All psychometric data were collected, and interviews conducted and transcribed by the first author. Four out of 10 interviews were rated by a second coder as a recommended measure of reliability [22].

### 2.3. Measures

The *Parental Development Interview on Reflective Functioning (PDI-RF)* [22] is a manualized assessment of RF in the form of a semi-structured interview. Mothers are asked about their relationship with their child, e.g., “Describe a time in the last week when you and your child really clicked”, the affective experience of parenting, e.g., “How has having a child changed you?” and the parent’s family history, e.g., “How do you think your experiences being parented affect your experience of being a parent now?”. The PDI-RF is based on four dimensions with scores ranging from 1 (negative RF) to 9 (exceptional RF). These four dimensions are: (1) the awareness of the nature of mental states, (2) the explicit effort to tease out mental states underlying behavior, (3) the recognition of developmental aspects of mental states, and (4) mental states in relation to the interviewer. Prompts are also provided in the PDI-RF manual.

The *Hopkins Symptom Checklist (HSCL)* [23] is an established and widely used screening instrument to measure severe emotional distress and depression. While the HSCL does not supply a diagnosis, it allows the clinician to recognize symptoms universally associated with anxiety and depression. The first 10 items of this symptom inventory relate to anxiety, and the remaining 15 items measure depressive symptoms. Each question asks about the frequency of specific symptoms in the past week using a 4-point frequency scale, where 1 = not at all and 4 = extremely. The average of the item score will result in a total score for depression, anxiety, and distress. Patients with scores of greater than 1.75 on anxiety and/or depression are considered symptomatic.

The *PSTD Checklist (PCL-5)* [24] assesses DSM-5 symptoms of posttraumatic stress disorder (PTSD). A total symptom severity score (range: 0-80) can be obtained by summing the scores for each of the 20 items score on a Likert scale of 0–4. DSM-5 symptom cluster severity scores can be obtained by summing the scores for the items within a given cluster, i.e., cluster B (items 1–5), cluster C (items 6–7), cluster D (items 8–14), and cluster E (items 15–20). A provisional PTSD diagnosis can be made by treating each item rated as 2 = “moderately” or higher as an endorsed symptom, and then following the DSM-5 diagnostic rule which requires at least 1 B item, 1 C item, 2 D items, and 2 E items. This scale has been used successfully among refugees residing in Europe [25].

The *Difficulties in Emotion Regulation Scale (DERS)* [26] was designed to assess multiple aspects of emotion dysregulation. The measure yields a total score, as well as scores on six scales derived through factor analysis: (1) non-acceptance of emotional responses, (2) difficulties engaging in goal-directed behavior, (3) impulse control difficulties, (4) lack of emotional awareness, (5) limited access to emotion regulation strategies, and (6) lack of emotional clarity. The DERS consists of 36 items scored on a 5-point Likert scale ranging from “almost never” to “almost always”.

The *Conner–Davidson Resilience Scale (CD-RISC)* [27] comprises 25 items, each rated on a 5-point scale (0–4) ranging from 0 (“not true at all”) to 4 (“true nearly all of the time”), with total scores ranging from 0 to 100. Higher scores reflect greater resilience. In this scale, scores between 0 and 49 are considered low, scores between 50 and 79 are considered medium, and scores from 80 to 100 represent a high resilience level. The CD-RISC has been used successfully in refugee populations [28].

### 2.4. Data Analysis

#### 2.4.1. Scoring PDI-RF Interviews

Scoring on the PDI-RF was calculated in two ways. Using knowledge from the training and the PDI-RF manual as a guide [22], first the response to each question was coded from 1 (negative RF) to 9 (exceptional RF). Then, using the individual scores as a reference, an overall score for RF on the basis of the interview as a whole was derived. A second coder then reviewed four interviews as a measure of reliability. Any discrepancies were then discussed and re-coded if necessary. Upon agreement, it was this overall score that was used as the measure of RF.

#### 2.4.2. Qualitative Analysis

In this study, the discourse from PDI-RF interviews was then used for qualitative analysis. After overall scores were calculated for PDI-RF interviews, the narrative discourse generated was analyzed using principles of thematic analysis [29]. The PDI-RF transcripts were first reviewed to allow initial prominent patterns to emerge. These were then refined into key themes, which allowed for the identification of overarching narratives. Secondly, on the basis of comparing the experiences of mothers with CBSV and those without, different elements featuring in the narratives of their relationship to their children, that may indeed impact their parenting, were explored. Coding was conducted in NVivo, v11.4.3 (QSR International, 2018).

Despite the small sample size, demographic characteristics and psychometric measures were statistically analyzed to identify emerging trends and give context to the population and act as a basis for future investigation. Testing of differences on mean age and length of stay in the Netherlands between both groups was done using Student’s *t*-test, and chi-squared tests were used for comparing categorical variables (asylum status, marital status, and religion). All analyses were performed with SPSS version 25 (IBM, 2018).

## 3. Results

Mothers in this study originated from six different countries: Armenia, the Democratic Republic of Congo, Eritrea, Iraq, Sierra Leone, and Uganda. Both mothers and children in the CBSV group of this study were younger, and these mothers scored significantly lower on the measure of parental RF (*t* = –3.54, *p* = 0.24). As there were no significant differences in symptoms of PTSD and depression or difficulties in emotion regulation (mothers in the war-affected group even tended to score higher), the lower level of reflective functioning in mothers with CBSV cannot be explained by these symptoms. The demographic characteristics and psychometric results can be seen in Table 1.

As per our research objective, part of the analysis was to discern the capacities of mothers with CBSV as compared with refugee mothers without CBSV. This was followed by findings on a wider level that apply largely to all mothers in such a sample.

### 3.1. Parenting in the Context of CBSV

Reflective functioning scores for mothers with CBSV were in the low range (average score of three out of a possible nine), indicating that they were unable to explicitly connect behaviors and emotions between themselves and their child. The language they used to discuss this relationship also failed to go beyond very basic expressions of mental states and was in stark contrast to other mothers in the wider sample, of whom some were markedly articulate. As one mother with a CBSV described: “The last time I was angry I was shouting. I was crying, very, very angry. What can help is when I shout. When I shout a lot, it helps me. I don’t get aggressive to my children—I don’t beat nobody. But I can like, the TV is going off, no tablet, no phone and I keep shouting. If I shout more, I can be released, and I can go on.” This mother demonstrated that she was aware of the need to process her negative emotions, but the way in which she described managing situations when she was angry is likely something that may have prevented her from being able to reflect on how this might impact her child. She went on to explain that when her child hears that she is no longer shouting, the child comes to apologize. In this instance, it appeared more difficult for her to access abstract thoughts concerning the mental states of her child. It is possible that this mother was unable to grasp the complexity of her child’s emotions and behaviors, and how her own behavior may be received by her children. Again, this was not the case with mothers in the war-affected group; they frequently made attempts to understand the interactional process and their children’s needs underlying their outward behavior.

When prompted to consider the interactional sequence of mental states and behaviors, mothers with CBSV frequently indicated they had “no idea” or had “never been asked” such questions. A core element of RF that can be identified during the interview is not that parents must be totally sure of the reason for their child’s behavior, but that those who make an attempt to understand score more highly. Responses such as these suggest that mothers were not actively attempting to discern the child’s needs underlying the outward behavior, and hence scored lower. Relatedly, mothers with CBSV were also unable to reflect on other complex negative emotions of their own such as guilt or feeling “needy”. Often these questions were shut down with simple “no” answers, or genuine surprise that any mother would feel such things. The lack of willingness to explore these types of mental states again meant that mothers scored lower.

Furthermore, mothers with CBSV all described crying in the presence of their children at times when they felt overwhelmed by the responsibility of parenting: “I was crying all the time because she was really sick: she had a bad cold and fever, vomiting all the time. It was really a hard time for me”, or with managing their emotions: “she senses most when I’m crying. She loves coming next to me and gives me a hug. At times I’m really scared because it may affect her, which I don’t want”. This may be as a result of capacities for emotion regulation that are impacted by traumatic events such as CRSV. Perhaps the interpersonal trauma experienced by mothers who are now raising CBSV prevents them regulating their own, and therefore also their child’s, emotions.

Moreover, mothers with CBSV expressed their ambivalence towards parenting, and some of their changing emotions over time: “I’m raising her. It’s something really, really, really difficult. But I’m doing it. I don’t regret it anymore. I won’t lie I used to, but I don’t anymore”. Four mothers explained this ambivalence as arising since they were now the sole caregiver for their child(ren). These mothers are raising children they did not plan to have, and they are now displaced from their home which in many cultures, means they miss out on the support of wider family members who would normally assist with childcare. In the words of one mother, they: “play the role of mother and father”. Such mothers described both acceptance of the situation as it is now, but equally how their being together with their child all the time can be overwhelming. One mother shared that she had never been apart from her child since the day she was born. This can, in some cases, lead to over-connected mother–child relationships and enmeshment that may prevent a mother being able to discern between her own mental states and those of her child. As one mother said: “When she sees me crying, she hugs me…she tries to wipe my tears then, she tries to hug me, yeah she loves that”. In these situations, it was likely that the mother was confusing her own emotions with those of her child, which would lead to lower RF scores. This kind of behavior could also be explained by insecure attachment patterns; the ability to reflect on the relationship with one’s child is based on the attachment patterns of one’s own childhood.

All mothers with CBSV in this sample shared that they were raised by single mothers (or in one case a grandmother) without father figures present in their lives, which may have served to create insecure attachment patterns. The absence of a father figure may be particularly poignant for mothers whose own children were conceived in the event of CRSV and will also grow up without the presence of their biological father, as compared to mothers in the control group who all talked about growing up with both their parents in the home. These findings could also be related to a reversal of roles that has been observed in other vulnerable mother–child dyads [30], where it is the child who increasingly provides the mother with emotional support. This could be a risk factor for the development of disorganized attachment relationships.

### 3.2. Wider Findings

While there are factors impacting parenting that can be uniquely attributed to mothers raising a CBSV, mothers across the whole study sample discussed factors in common: mental well-being, the universal challenges of motherhood, or indeed difficulties that pertained to their status as refugees.

Not all mothers in this sample had experienced CRSV; however, they had all experienced some type of trauma and were managing their mental well-being as a result. At times this reflected in their descriptions of parenting. One mother felt a sense of guilt for bringing her daughter into the world during a coercive relationship with the child’s father. She fled this, as well as war in her country, but explained it as something she wanted to protect her child from knowing about: “it’s a kind of secret that I keep in myself”. This mother explained that seeing a psychologist regularly was the only way she managed to process her past, so that it did not impact on her relationship with her daughter. Seven other mothers in this study were also currently in therapy, with one more having seen a psychologist in the past. During the interviews, mothers talked about how their therapy helped to regulate their emotions and manage their deteriorating mental health: “about one and half years ago I had no voice anymore, because every time that I get angry, I began to scream. After some therapies and trying to keep my voice inside, and not starting to scream more, now I get through that moment by crying.” Equally, mothers in this study talked about coping, both in relation to their cultural background and how they tried to pass it onto their children, so that they could grow up in the same way. Mothers with and without CBSV also shared how being a mother in itself was empowering, and how this was their source of strength: “[My child] is my motivation in life. [My child] gives me purpose in life, [they] give me an idea of the future, of what will happen tomorrow. I think all mothers feel this way.” Mothers able to identify and articulate this sense of purpose as a mother had higher RF scores.

The universal challenges of parenthood—for example, managing daily routines and navigating tantrums or bad behavior—were explicitly mentioned by 5/10 mothers in this study. When describing these sorts of situations, one mother said: “I want her to know, being a child, somethings you can do and somethings you cannot do. And I am her mother so sometimes she has to accept that I decide it for her.” Seven mothers, however, reflected upon their status in the Netherlands as refugees or asylum seekers, and how their current or past living situations interacted with their ability to parent. This theme played a central role in narratives of mothers in this study. Upon arriving in the Netherlands, 2 mothers in this study were with their children and other family members, 5 arrived with only their child(ren), 1 was separated from her children for several years upon arrival, 1 gave birth to her child while in immigration detention, and 1 gave birth to her child in a Dutch asylum center. One mother described how being apart from her children for some years in a new country impacted their relationship: “she would ask me ‘Mama why are we not together?’ I would have to explain why we are not together; I would like to be together, but it feels very dishonest that we cannot.” This was also the case for mothers who escaped conflict or unrest in their country and made dangerous migration journeys. One mother recalled the stress this caused during her pregnancy: “When I came to know that I was pregnant, oh I was so happy (…), I was really dreaming about it. On the same day I had an appointment with the police to discuss my stay permit. I went to this appointment and they kept me there, they took me to prison. When I came out of prison, they withdrew my stay permit.” Seven mothers explained that these were not optimal situations in which to raise their child(ren): that asylum centers and shelters did not provide space or privacy, meaning that they could not make the decisions they would like to in order to discipline their children, and that their surroundings meant—even when children attended local schools—that their upbringings were different and not like those of many other children: “I realize that there are so many borders for us, there are so many issues that I cannot take care of like a parent should. We cannot take the decisions that we need.”

Five mothers without permits (both at the time of the study and in the past before they were granted refugee status) also talked about their uncertainty regarding the arduous asylum process: “it’s long procedure, so if they give you negative, they throw you out. You have to either go back to your country or…find another solution to help you”, but none spoke of wanting to return to their native countries. Two mothers talked about wanting to adopt their new culture, while at the same time maintaining connection to their heritage, and how this affected their interactions with their children: “my culture is different from the Dutch. Here they grow up in a different country. I want them to know my religion. I want them to be respectful like in my culture—you have to be respectful to older people, than people who are the same age as you. The freedom they have here is different from my home”.

Mothers also shared their experiences of isolation as a result of having little social support, especially when they were provided housing outside of major cities with limited cultural diversity. One mother shared how she was one of only a few women of color in her neighborhood and described being stopped by men in cars asking for sex while out with her child. Moreover, all mothers in this sample shared they actively practiced religion, but none were able to engage with their faith communities since it was not feasible in their situations (travel costs, distance, etc.). Equally, two other mothers talked about their difficulty in attending appointments or language classes (both compulsory for official status in the Netherlands) when they had no-one to care for their children.

## 4. Discussion

This study explored reflective functioning in trauma-affected mothers now residing as refugees in the Netherlands, both with and without a CBSV. We hypothesized that mothers with CBSV would have lower RF scores than other mothers, which our results reflected. Specifically, findings from this study indicate that in general, this sample of refugee mothers in the Netherlands has levels of reflective functioning similar to those seen in prior PDI-RF interviews conducted within the general population [6]. This means these mothers are able to make explicit mentalizations and reflections of mental states that are not cliché, and that the mother has a model of mind of herself and child. When analyzed independently, mothers who had been granted refugee status and asylum seekers in the Netherlands maintained ordinary levels of RF. However, mothers with CBSV had lower scores, indicating they were unable to extend their reflections of mental states towards more sophisticated responses that comprised complex emotions; emotions that were unusual or painful or interpreted as an interactional sequence between the mother and child. This finding is consistent in mothers with histories of violence and poverty [31] and might reflect the extreme vulnerability of these two particular groups since their emotional burden extends beyond that of mothers granted the official right to remain in the Netherlands, as they face huge amounts of uncertainty.

The findings of this study correspond with those of other research studies in the field of attachment in populations of refugees, where it was found that mothers with experiences pertaining to conflict and migration (e.g., torture, threats of execution, forced separation from family members) displayed ambivalence towards their children [18]. Some mothers in this study shared their oscillating feelings during their pregnancy or their child’s life, and it is possible this affected their ability to make sense of a child’s behavior and accurately respond to their needs; if a mother is particularly overwhelmed with distress that relates to the conception of her child, she might not see this child in a favorable light and thus not judge their needs objectively. In the present sample, particularly for mothers with CBSV, mothers also described feelings of ambivalence towards having a child conceived without their consent or planning for it, in line with data gathered from specialists working in the field of CRSV who have observed similar reactions [32].

Mothers also described universal experiences of parenting that do not pertain to their being a refugee, such as being a single parent. However, because of their situation they were not able to parent as effectively as they would have liked since asylum centers and associated formalities prevented typical interactions. This included having not enough privacy or a separate space for use in “time-out” for a child, or not having their child engage with peers in the same way they might have done if they lived outside the asylum center. This reflects similar findings that the daily stresses associated with being a refugee [33] can negatively impact the level of parenting sensitivity [11,12].

Community connectedness appears to play important role in the lives of refugee mothers and children. Women in this study shared on the lack of social interaction (e.g., religious, with peers, intimately) that often occurred when they were provided housing in rural areas. While having their own space in a house was welcomed, they often missed out on attending events since they did not have access to childcare, for example. Thus, feelings of social isolation likely increase as a result of having a limited support system in the Netherlands, both while seeking asylum (or residing without papers), and after receiving official refugee status. Particularly for mothers with CBSV, it was not unusual for them to spend all their time together—some every day since the child’s birth. Over-connected mother–child relationships run the risk of become enmeshed, particularly when mothers themselves have conflicting existing attachment patterns [34]. Enmeshed behaviors become central to relationships when RF capacities are already low [35] and may cause mothers not to recognize their child’s needs as separate from their own, perhaps resulting in lower parental sensitivity. Some questions in the PDI interview required abstract thought and reflections of emotions, and mothers with CBSV in this sample had more difficulty in making these connections; they had seemingly never before considered the interactional process by which their relationship might affect their child’s personality and development. As such, the language used in responses to abstract questions was oftentimes very limited and took much prompting to reach an answer. This could be related to several factors, for example education (corresponding with previous evidence that suggests lower RF is associated with lower levels of education, [36]); the clarity of memory recall (which may be altered for events appraised as traumatic [37]); or their own insecure attachment patterns from their childhood (which are brought forward into their relationship with their child [38,39]). Indeed, this could also be related to the detrimental impact that maternal psychopathology has on parenting capacities [40,41], in that limited access to emotional regulation impacts a parent’s ability to function and parent effectively. Untangling this complex interplay necessitates further research.

### 4.1. Limitations

There are some clear limitations to this study, and results should consequently be interpreted with caution. Owing to the circumstances in which to recruit women to this study—the sensitivity of such a topic, the time necessary to make contact and gain the trust of these women, and the often chaotic lives of refugees—the sample was small and quantitative results are to be used as a guideline only. They ought not be used to generalize vastly across refugees or mothers with CBSV. Furthermore, the qualitative analysis conducted on data collected during the semi-structured PDI-RF interview in this study was somewhat limited since it was not open-ended and did not have the original purpose of broadly addressing parenting experiences. In addition, during one interview the participant’s husband was present and responded to questions when the mother became overwhelmed. While this was useful in understanding their situation as refugees, despite lengths being taken to not transcribe or code responses by the husband, the mother’s narrative may have been impacted. Furthermore, eight interviews were conducted using interpreters. Many of these expressed that some words did not have a direct translation, and they therefore had to use similar words or phrases to convey the same message, opening up a possibility for misinterpretation and inconsistencies. Moreover, this study was carried out while refugees lived in relative safety, and not still in proximity of the threat of war and traumatic reminders [12]; thus, these results must be interpreted with this in mind, and further research should extend the investigation of mothers and CBSV to local contexts and those of internal displacement. These limitations notwithstanding, failure to utilize the discourse obtained through the PDI-RF interviews and the small amount of quantitative results as a baseline for such a vulnerable group would have been an oversight and a waste of valuable information.

### 4.2. Recommendations

The results of this study can act as a basis for future work and serve to identify preliminary data trends and patterns of functioning within a very specific population that would benefit from further sensitive exploration. Lower levels of RF in this sample suggest that mothers with CBSV are less able to articulate concepts of mentalization and complex mental states than other mothers. This notion warrants future research in order to add weight to the findings of this study. Nonetheless, clinicians should be aware of the possibility that RF may be affected in these populations. Future programming and supportive work are recommended to address the trauma faced by women who have experienced sexual violence, enable mothers to learn to be reflective [42], and help them with parenting difficulties and making sense of their relationships. This element is of particular importance in light of the way in which the children of some women are conceived. Lastly, often the tendency is to look at what is not working or what brings people to seek help, but strength and growth are possible, and many women (and families) find solace in the spaces and services offered to asylum seekers and refugees. Taking a proactive approach in supporting these mothers, as opposed to waiting until they require specialist services, could be useful in addressing social isolation and integration, and possible mental ill-health.

## 5. Conclusions

This study is the first of its kind to assess RF in a sample of refugee women in the Netherlands, some of whom had experienced CRSV and were raising a child as a result of this. Mothers overall tended towards reflective functioning capacities similar to those of the general population and mothers without trauma or migration histories. Mothers raising CBSV were found to have lower reflective functioning abilities, similar to those seen in populations with trauma or violent histories. These mothers appeared to be impacted by emotion dysregulation and ambivalence, which likely impacted their relationships with their children. At a wider level, these trends suggest that the parenting capacity of women in this study (irrespective of how their child was conceived) was impacted by their being refugees. While it very often offers stability and a safe space, these preliminary findings suggest that the asylum process could possibly act as a ceiling to the degree of recovery that can be achieved. An expanded study investigating RF among refugee women would add weight to the trends identified in this small study.

## Figures and Tables

**Table 1:** Demographic characteristics and psychometric scores of the study sample.

	Total (*n* = 10)		Mothers with CBSV (*n* = 5)		War-affected Mothers (*n* = 5)		
	Mean	SD	Mean	SD	Mean	SD	Statistical Test
**Age**	31.2	5.87	27.6	6.27	34.8	2.39	*t* (8) = −2.44*
**Age of Child**	4.6	3.27	2.6	3.05	6.6	2.19	*t* (8) = −2.38*
**Length of Stay in NL**	5.7	4.22	4.8	5.93	6.6	1.67	*t* (8) = −0.65
	**Frequency**	**(%)**	**Frequency**	**(%)**	**Frequency**	**(%)**	
**Asylum Status**							*χ^2^* (2) = 2.53
Refugee	5	50	2	20	3	30	
Asylum seeker	3	30	1	10	2	20	
Undocumented	2	20	2	20	0	0	
**Marital Status**							*χ^2^* (2) = 4.80
Married	5	50	1	10	4	40	
Single	3	30	3	30	0	0	
Divorced	2	20	1	10	1	10	
**Religion**							*χ^2^* (2) = 3.00
Muslim	4	40	2	20	2	20	
Catholic	2	20	2	20	0	0	
Christian	4	40	1	10	3	30	
**Study Variables**	**Mean**	**SD**	**Mean**	**SD**	**Mean**	**SD**	
**PCL-5**	48.00	17.58	42.00	22.51	55.50	3.70	*t* (4) = −1.32
Cluster B	13.11	5.78	11.40	3.70	15.25	1.71	*t* (4) = −1.11
Cluster C	4.88	2.45	3.60	2.51	6.50	1.30	*t* (7) = −2.24
Cluster D	17.11	5.51	15.80	7.16	18.75	2.50	*t* (7) = −0.77
Cluster E	11.88	5.23	9.80	6.18	14.50	2.38	*t* (7) = −1.42
**HSCL**	75.78	14.31	69.20	15.12	84.00	8.86	*t* (7) = −1.72
Anxiety	2.74	0.85	2.40	1.00	3.18	0.39	*t* (7) = −1.45
Depression	3.23	0.61	3.02	0.67	3.50	0.48	*t* (7) = −1.20
**DERS**	110.78	31.74	98.80	37.80	125.75	15.61	*t* (7) = −1.32
Non-acceptance	17.33	7.03	16.00	8.75	19.00	4.83	*t* (7) = −0.61
Goals	18.33	5.96	15.60	6.80	21.75	2.22	*t* (7) = −1.72
Impulse control	18.89	8.81	14.40	8.32	24.50	6.25	*t* (7) = −2.01
Awareness	15.00	4.63	15.00	5.74	15.00	3.65	*t* (7) = 0.00
Strategies	25.78	7.38	22.40	6.46	30.00	6.83	*t* (7) = −1.71
Clarity	12.56	4.56	12.00	6.12	13.25	2.06	*t* (7) = −0.43
**CD-RISC**	55.11	20.91	55.40	21.81	54.75	23.04	*t* (7) = 0.043
**PDI-RF Score**	4.5	1.65	3.00	0.00	5.40	1.52	*t* (7) = −3.54*

* *p* = < 0.05. PCL-5: Posttraumatic checklist. HSCL: Hopkins Symptom checklist. DERS: Difficulties in Emotion Regulation Scale. CD-RISC: Connor–Davidson Resilience Scale. PDI-RF: Parental Development Interview for Reflective Functioning.
